# Supplementary material for: Composite Probiotics Improve Gut Health and Enhance Tryptophan Metabolism in Nursery Piglets During Liquid Feeding
Source: Int J Mol Sci. 2025 Jun 13;26(12):5698. doi: 10.3390/ijms26125698 (PMC12192887; doi:10.3390/ijms26125698)
Supplement: Supplementary file 1 [file ijms-26-05698-s001.zip › ijms-3664040-supplementary.pdf]

Composite Probiotics Improve Gut Health and Enhance Tryptophan Metabolism in Nursery Piglets during Liquid Feeding

Man Du [1, 2, 3], Qifan Zhang [1, 2, 3], Yutian Shen [1, 2, 3], Jie Fu [1, 2, 3], Yizhen Wang [1, 2, 3], Bin Yao [4], Zeqing Lu [1, 2, 3, *]

[1] National Engineering Research Center of Green Feeds and Healthy Livestock Industry, Zhejiang University, 866 Yuhang Tang Road, Hangzhou, Zhejiang 310058, China.

[2] Key Laboratory of Animal Nutrition and Feed, Ministry of Agricultural and Rural Affairs, Zhejiang University, 866 Yuhang Tang Road, Hangzhou, Zhejiang 310058, China.

[3] Key Laboratory of Molecular Animal Nutrition, Ministry of Education, Zhejiang University, 866 Yuhang Tang Road, Hangzhou, Zhejiang 310058, China.

[4] State Key Laboratory of Animal Nutrition and Feeding, Institute of Animal Science, Chinese Academy of Agricultural Sciences, Beijing 100193, China

*Corresponding author.

Email address: zqlu2012@zju.edu.cn (Zeqing Lu).

[1] Present address:

College of Animal Sciences, Zhejiang University, Hangzhou, China

Supplementary figures

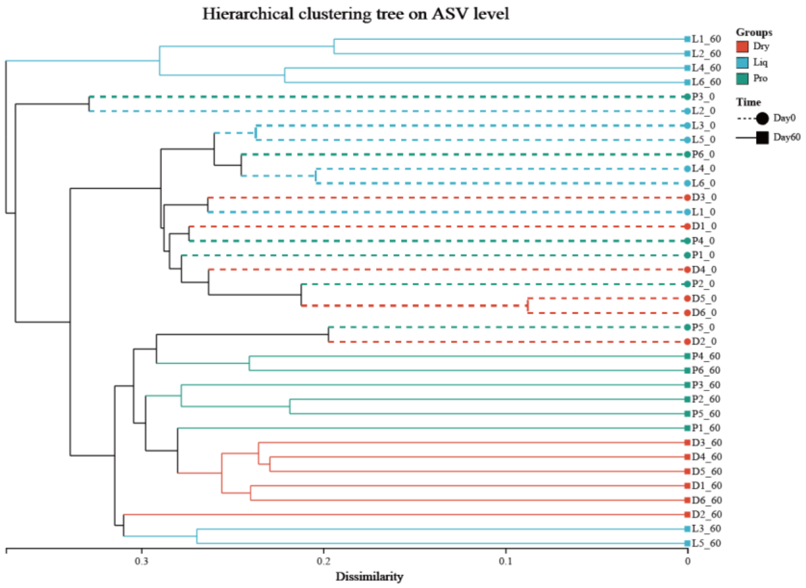

Figure S1. Supplementary analyses related to Figure 3.

Hierarchical clustering of bacterial community composition based on Bray-Curtis distances. Dry = solid feed group; Liq = liquid feed group; Pro = probiotic-enriched liquid feed group; Day 0 = start of experiment; Day 60 = end of experiment.

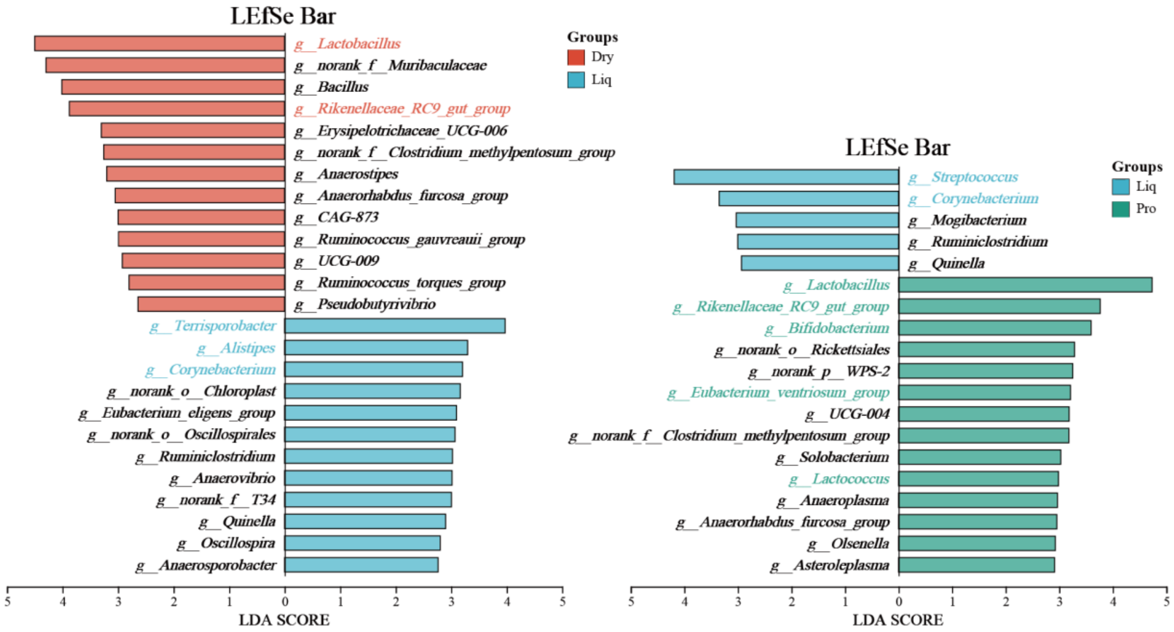

Figure S2. Supplementary analyses related to Figure 4.

Lefse analysis for microbial differences between Dry and Liq or Liq and Pro groups.

Dry = solid feed group; Liq = liquid feed group; Pro = probiotic-enriched liquid feed group; Day 0 = start of experiment; Day 60 = end of experiment.

Supplementary tables

Table S1. Composition of inorganic salt medium.

**Ingredient**	**Addition amount (%)**
$NaHCO_3$	0.4
Cysteine	0.1
K_2HPO_4	0.045
KH_2PO_4	0.045
NaCl	0.09
$MgSO_4{\cdot}7H_2O$	0.009
$CaCl_2$	0.009
Haemin	0.001
Vitamin Supplement	1
Mineral Supplement	1

$MgSO_4$ and $CaCl_2$ were sterilized separately prior to use. After combining the sterilized components, the mixture was allowed to cool to room temperature. Subsequently, tryptophan, vitamin supplements, and mineral supplements were added sequentially. The final solution was then filtered through a 0.22 μm membrane to remove any potential microbial contaminants and particulates, thereby yielding a sterile inorganic salt medium.

Table S2. PERMANOVA test for effects of age and group on differences in microbial composition.

**Name**	**Df**	**SumsOfSqs**	**MeanSqs**	**F.Models**	**R2**	**Pr(>F)**
Age	1	1.442053	1.442053	7.833722	0.18351	0.001
Group	2	0.525456	0.262728	1.427229	0.066868	0.06
Residuals	32	5.890648	0.184083	-	0.749622	-
Total	35	7.858157	-	-	1	-

Table S3. PERMANOVA test for effects of feed type and probiotic on differences in microbial composition.

**Name**	**Df**	**SumsOfSqs**	**MeanSqs**	**F.Models**	**R2**	**Pr(>F)**
Probiotic	1	0.403389	0.403389	2.346547	0.121881	0.002
Feed type	1	0.327698	0.327698	1.906244	0.099011	0.007
Residuals	15	2.578612	0.171907	-	0.779108	-
Total	17	3.309699	-	-	1	-
